# Generalizability and performance of methods to detect non-wear with free-living accelerometer recordings

**DOI:** 10.1038/s41598-023-29666-x

**Published:** 2023-02-13

**Authors:** Esben Lykke Skovgaard, Malthe Andreas Roswall, Natascha Holbæk Pedersen, Kristian Traberg Larsen, Anders Grøntved, Jan Christian Brønd

**Affiliations:** Research Unit for Exercise Epidemiology, Department of Sports Science and Clinical Biomechanics, Centre of Research in Childhood Health, University of Southern Denmark, 5230 Odense, Denmark

**Keywords:** Epidemiology, Machine learning

## Abstract

Wearable physical activity sensors are widely used in research and practice as they provide objective measures of human behavior at a low cost. An important challenge for accurate assessment of physical activity behavior in free-living is the detection non-wear. Traditionally, heuristic algorithms that rely on specific interval lengths have been employed to detect non-wear time; however, machine learned models are emerging. We explore the potential of detecting non-wear using decision trees that combine raw acceleration and skin temperature, and we investigate the generalizability of our models, traditional heuristic algorithms, and recently developed machine learned models by external validation. The Decision tree models were trained using one week of data from thigh- and hip-worn accelerometers from 64 children. External validation was performed using data from wrist-worn accelerometers of 42 adolescents. For non-wear episodes longer than 60 min, the heuristic algorithms performed the best with F1-scores above 0.96. However, regarding episodes shorter than 60 min, the best performing method was the decision tree model including the six most important predictors with F1 scores above 0.74 for all sensor locations. We conclude that for classifying non-wear time, researchers should carefully select an appropriate method and we encourage the use of external validation when reporting on machine learned non-wear models.

## Introduction

Over the past few decades body-worn motion sensors have been used to study human physical activity behavior as these devices have been shown to provide a robust method for measuring the characteristics of free-living movement^1–3^. The use of activity monitors based on accelerometry offers objective data capture and the ability to assess intensity of movement with minimal participant burden and at high cost efficiency^4^. Different methods are used to attach the devices on the subject and many protocols allow the subject to detach the device for water activities, sleep, or sports for which wearing the device can cause an injury. Non-wear time is defined as periods of not wearing the devices which can have important consequences for the outcomes derived from the acceleration measurements. Since non-wear time is equivalent to missing data, researchers may exclude the non-wear time from their analysis or perform imputation of the missing data using various methods such as zero-inflated Poisson and Log-normal distributions^5^. However, imputing non-wear time can introduce bias, especially for longer periods of non-wear time, because every imputation method is based on assumptions about the data, which may not be accurate. Therefore, the optimal classification and handling of these non-wear periods is important for providing researchers with high quality estimates of the subject’s physical activity behavior during free-living.

The classification of non-wear periods in the physical activity measurements can be obtained by having the subjects keep an individual log diary although this method is cumbersome for the subject and is potentially error prone^6^. To reduce the burden on the subject and increase accuracy, researchers have employed different rule-based methods and more advanced algorithms to classify non-wear time. The earliest rule-based methods were developed for ActiGraph counts data which classify non-wear time as the periods of data with consecutive zero counts exceeding a specified duration^7–9^. Although the simplicity of these methods is considered a strength, it has been shown that alternating the length of the time interval of consecutive zero counts result in differences in physical activity and sedentary behavior aggregates of up to 10%^10^. Moreover, Until recently, the algorithms used to convert acceleration data into counts were proprietary, which would impede the transparency of the research field. Finally, wear-time inclusion criteria have been shown to vary depending on non-wear settings with age and obesity level eliciting relative differences in non-wear time^11^. This makes the consecutive zeros algorithms sub-optimal and hinders comparisons across studies with differing populations and non-wear settings^11^.

The technological advances during the last couple of decades of accelerometers have provided researchers with the ability to store raw accelerations (in gravity units*),* which increases the granularity of the data and potentially the ability to accurately classify non-wear periods. Different algorithms have been developed to detect non-wear time in raw accelerometer data which typically utilize a standard deviation threshold but also in combination with surface skin temperature^14–16^. These heuristic approaches have proven to be generalizable across different populations, device brands and wear-sites, but having access to data of increasing quantity and quality does in principle not improve their performance as is possible with a machine learning approach which can improve as data becomes available. Furthermore, the use of simple duration-based algorithms carries a risk of falsely misclassifying true non-wear as inactivity as they are restricted by time length-specific intervals and any non-wear episodes shorter than the interval cannot be detected. In the current study, we define *generalizability* as the extent to which the performance of a model can be applied to out-of-sample unseen data, i.e., data beyond the specific sample used in the development of the model. If a model has low generalizability, its findings may be limited in their applicability to other populations or settings.

Recently, studies have investigated the potential of classifying non-wear using raw accelerometer data in conjunction with machine learning like random forests^17^ and deep learning techniques^18^. The purpose of using machine learning algorithms is to learn patterns from the training data and to approximate the complex model which best describes the relationship between the included predictors and the outcome. In this process, the trade-off between model variance and bias must be taken into consideration. Variance refers to the amount by which the approximated function would change if it were estimated on a different training dataset and bias refers to the error that is introduced by approximating a complicated problem with a model that assumes a simple relationship (e.g., linear regression estimating a highly non-linear function). Thus, high bias results in underfitting of the model and high variance results in overfitting. In general, highly flexible, and complex models with high variance/low bias pay a lot of attention to the training data and fail to generalize to unseen data. As a result, such models perform very well on the training data but have high prediction error on unseen data, i.e., the model is overfitted to the training data. Contrary, simpler models with less flexibility are prone to underfit the training data due to high bias and low variance. Therefore, the balance between overfitting and underfitting is of foremost importance when the predictive performance of a machine learned model is to be used on an unseen data source. Although the models utilizing complex machine learning algorithms have shown to perform exceptional on testing data, it is unknown to which degree the models perform on external unseen data. Although this can be viewed as a shortcoming of studies reporting performance metrics with no external validation, it is important to recognize that the performance of a given machine learning or deep learning model will always, in principle, be unknown on out-of-distribution data sources. However, as most non-wear detection methods are developed with the goal in mind of being device, placement, and population agnostic, external validation will be of value as an indication of model generalization. Despite this, there exists several reasons to why researchers are not employing external validation, including lack of out-of-distribution data sources and/or the desire to incorporate all available data into the training of the models to capture the most information. A few studies have been utilizing surface skin temperature in combination with raw acceleration for the classification of non-wear episodes^14,16^. However, the performance and generalizability of adding the surface skin temperature for the classification of non-wear with advanced machine learning methods has not been investigated.

To date, accurate classification of non-wear time in raw accelerometer data still has potential for improvements despite advancements in sensor technology and related software. This begs the question, as a tool for the essential first step when analyzing accelerometer data, i.e., classifying non-wear time, what heuristic algorithm or machine learned model will perform the best on unseen data?. To answer this, we created three datasets of raw accelerometer data with correctly labeled wear- and non-wear time as ground truth including surface skin temperature measurements. In specific, we aimed to (1) train three decision tree models on accelerometer data from thigh and hip-worn accelerometers for the classification of non-wear time in raw accelerometer data and evaluate the importance of surface skin temperature and minimizing the number of predictors provided to the model and (2) evaluate the performance of machine-learned models and simple heuristic algorithms across datasets of varying age ranges for the classification of non-wear time in raw accelerometer data.

## Background

We included in total four additional non-wear classification methods to evaluate generalizability and to compare the performance with the three new algorithms developed. These existing methods are carefully selected on the premise that we wished to examine a spectrum of method flexibility such that the simplest (and most widely used) and the most recent and complex techniques are included.

### Consecutive zeros-algorithm (*cz*_60)

A variety of consecutive zero-algorithms for count-based accelerometer data have been developed over the years for detecting periods of non-wear within specified time intervals, such as 30-, 60-, or 90-min intervals^7,9,19^. Furthermore, non-wear algorithms using raw acceleration have been developed by van Hees and colleagues with a 30 min interval^12^ who later extended their work with a 60-min interval algorith^15^, and one with a 135-min interval and tuned hyperparameters by Syed et al.^20^ Here we employ a simple implementation of this concept of detecting no movement based on Actigraphy counts with an algorithm that detects only zero counts for a minimum of 60 consecutive minutes. Actigraphy counts are generated with a deadband of 68 mg, making it the minimum detectable acceleration threshold.

### Heuristic algorithm (*heu_alg*)

This algorithm is described in detail by Rasmussen and colleges^15^ and utilizes a combination of raw acceleration and surface skin temperature. Periods longer than 120 min with accelerations below 20 mg are always identified as non-wear time and periods between 45 and 120 min are identified as non-wear if the temperature is below an individually estimated non-moving temperature threshold. Finally, the algorithm detects non-wear periods of 10–45 min in duration only if the end of the non-wear period is within the expected awake time.

### Random forests model (*sunda_RF*)

The non-wear classification method described by Sundararajan et al. is based on a random forest ensemble model which was trained on raw accelerometer data from 134 subjects aged 20–70 years. The subjects wore an accelerometer on their wrist during a single overnight PSG recording. The ground truth labels for non-wear time assumed that the accelerometer was worn only during the PSG recording. Only in epochs where the standard deviation in the acceleration signal per 15 min was larger than 13.0 mg outside the PSG recording was labelled as wear time. As input data, 36 predictors were constructed, and a nested cross-validation approach was employed to obtain generalization performance and to tune hyperparameters.

### Deep convolutional neural network (*syed_CNN*)

The non-wear classification method described by Syed et al.^18^ is based on a deep convolutional neural network using a novel approach which is different from the other methods included. Initially, all candidate non-wear episodes are identified using a standard deviation threshold. Then, rather than inspecting acceleration within the candidate non-wear intervals as the other methods do, this approach examined the signal shape of the raw acceleration right before and right after an episode of non-wear time using a convolutional neural network (CNN). In essence, they developed a CNN that can infer non-wear time by detecting when the accelerometer is detached and when it is mounted back on again. In this study, we used a window length on each side of the candidate non-wear period of 10 s, as this produced the best results. The training data for developing the CNN was collected from hip-worn accelerometers of 583 participants aged 40–84 years (mean = 62.74; SD = 10.25).

## Methods

**Figure 1:**
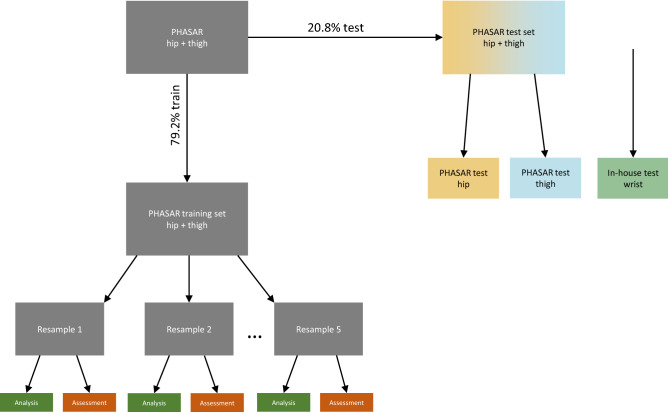
Flowchart of the splitting of the PHASAR dataset into train and test. The boxes on the left represent 79.2% of the PHASAR data for training in the five-fold resamples. The yellow and blue boxes on the right represent 20.2% of the PHASAR data for testing being split up into hip and thigh data while the green box is our in-house test dataset collected from wrist-worn devices.

Investigating the classification performance and generalizability of the non-wear classification methods was done by evaluating the performance with data collected in free-living using accelerometers placed at the wrist, thigh, and hip. The data was established by combining data collected in the Physical Activity in Schools After the Reform study (PHASAR)^21^ which provides data for both hip- and thigh, and an in-house data validation study which collected acceleration with wrist-worn accelerometers. An outline of how the data was used is presented in Fig. 1. Using the three data sets in this way, we ensured that the previously established non-wear classification methods were evaluated on an external data set. We also ensured that our own decision tree models were evaluated on an external independent dataset with data and wear location, which was not used for developing the decision tree models. Thus, all included machine learned models are evaluated with a test dataset collected from anatomical positions both included and not included in the development of the models.

### Data sources

With both the PHASAR and in-house validation study, raw acceleration data in conjunction with surface skin temperature was recorded by the Axivity AX3 accelerometer (Axivity Ltd., Newcastle upon Tyne, UK). The Axivity AX3 provides a dynamic range of ± 8 g (1 g = 9.81 ms^−2^), with physical dimensions 23 mm × 32.5 mm × 7.6 mm and weighing only 11 g. The Axivity AX3 device stores acceleration in gravity units (g) along three axes (vertical, mediolateral, and anteroposterior) with a selectable sampling frequency of 6.25–3200 Hz. The sampling frequency was set to 50 Hz with the PHASAR study and 25 Hz with the in-house validation study. All acceleration data was resampled into 30 Hz for both studies.

The PHASAR study is comprised of a population-based sample of more than 2000 school-aged children from 31 public schools in Denmark^21^. The objectively measured physical activity data from hip- and thigh-worn accelerometers were obtained in the PHASAR study during 2017–2018 and consisted of data from 1315 (49%) boys and 1,358 (51%) girls aged 8.1–17.9 years old (mean = 12.14, SD = 2.40). The accelerometers were worn at two anatomical locations; one fixated to the body in a pocket attached to a belt worn around the waist, where the sensor was placed on the right hip with the USB connector facing away from the right side of the body. A second belt was worn around the right thigh midway between the hip and the knee, where the accelerometer was placed in a pocket with the USB connector facing away from the body. The devices were worn for 1 week (seven consecutive days) which corresponds to the recommended number of days required to reliably estimate habitual physical activity^22^. The in-house validation study is comprised of accelerometer recordings from 42 individuals, 21 boys and 21 girls. This data was collected on youth athletes aged 14.5–16.4 years old (mean = 15.4, SD = 0.37 years) in the Region of Southern Denmark, which were all enrolled in a tailored talent program at two public schools near the end of their high school period. The Axivity accelerometer was mounted to the non-dominant wrist using a rubber strap with an embedded socket for the sensor and was worn for 14 consecutive days. The data collection for the in-house validation study commenced in the early spring of 2021.

From the PHASAR cohort we included the raw accelerometer recordings of 64 randomly selected participants. Based on this dataset of 64 participants, a dataset with labeled episodes of true non-wear time was constructed by means of manual annotation, a methodology, which is described in detail elsewhere^23^. In short, the non-wear episodes were inferred by visually inspecting the raw accelerations in combination with skin temperature readings. From raw triaxial 30 Hz Axivity acceleration data, episodes of true non-wear with start and stop time stamps were manually labelled in each of the three constructed datasets and served as labels of ground truth in later analyses. A second dataset was constructed in the exact same manner from the in-house validation study including all 42 youth athletes.

The PHASAR study was assessed by the Regional Committee on Health Research Ethics for Southern Denmark (ID: S-20170031) and deemed not eligible to undergo ethics review (documentation available on request to corresponding author) By Danish law, only research projects of biomedical character or projects that involve risks for participants need to have their ethics reviewed by a Regional Ethics Board. All other research projects can not apply for formal ethical approval. The in-house validation study was by the Research & Innovation Organization and legal department of University of Southern Denmark waived for ethical approval through the regional ethical committee. Both studies were carried out in accordance with the Danish Data Protection Agency (2015-57-0008) and all included participants, and/or their legal guardian(s) gave written informed consent. Furthermore, all methods were carried out in accordance with relevant guidelines and regulations (i.e., Declaration of Helsinki).

### Development of decision tree models

For the development of our decision tree models, we extracted a total of 12 predictors from the raw accelerometer data from the PHASAR datasets including temperature, time of day, integer indicator variables for device placement and day of week and moving average statistics (see Table 1). Moving average predictors were aggregated in 10 s epochs.

**Table 1:** Predictors derived from the raw sensor signals.

Predictor	Description
Weekday	Day of week ([1:7])
time_day	Time of day (milliseconds)
Location	Device wear location: 0 = thigh, 1 = hip
macc_x	Moving average of the z axis acceleration
macc_y	Moving average of the y axis acceleration
macc_z	Moving average of the z axis acceleration
sdacc_x	Moving average of the standard deviation on the x axis acceleration
sdacc_y	Moving average of the standard deviation of the y axis acceleration
sdacc_z	Moving average of the standard deviation of the z axis acceleration
Sdmax	Maximum standard deviation
Incl	Inclination angle of the device in relation to the direction of the gravitational force
Temp	Surface skin temperature (degrees Celsius)

The training of the decision tree models was performed on 79.2% of the PHASAR data stratified on non-wear and wear time including both the hip- and thigh-worn data (see Fig. 1. Furthermore, the data was split into training and test partitions such that participants in both partitions did not overlap which ensures that the algorithm did not learn any patterns specific to individual participant behavior. To optimize model hyperparameters and to minimize the risk of overfitting, a five-fold cross-validation scheme was used. The hyperparameters that were tuned were cost-complexity, tree depth and minimum number of data points required in a node for further splitting. For this, Latin hypercube sampling was used to construct a space-filling parameter grid with 10 levels that cover the parameter space such that any portion of the space had an observed combination that was not too far from it. The model with the most optimal hyperparameters was then trained on the full training dataset, still including both hip- and thigh-worn accelerometer data.

**Figure 2:**
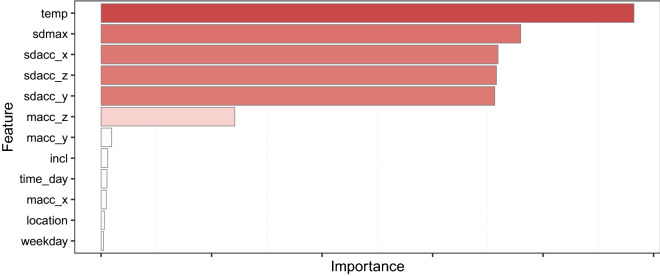
Predictor permutation importance plot for the decision tree including all predictors. The six most important predictors were used for training a second decision tree (*tree_imp6*), while all predictors excluding temperature were used to train a third decision tree (*tree_no_temp*).

In the present work we present a full model (*tree_full*) including all predictors, a second model including the six best predictors (*tree_imp6*) based on permutation predictor importance (see Fig. 2), and a third model excluding the surface skin temperature (*tree_no_temp*) resulting in 5 times 10 models trained in the process per decision tree variant. The slight imbalance in the outcome variable of 55.8% wear time vs 44.2% non-wear time did not warrant the need to apply synthetic minority oversampling techniques (i.e., SMOTE) or other techniques to balance out the data.

### Statistics

We calculated the classification performance to each ground truth test dataset comprising of more than 7 million 10 s epochs from 104 different subjects. True non-wear time inferred as non-wear time contributed to the true positives (TP), and true wear time inferred as wear time contributed to the true negatives (TN). Both TPs and TNs are required to obtain high accuracy of the non-wear time algorithm, as they are the correctly inferred classifications. True non-wear time inferred as wear time contributed to the false negatives (FN), and true wear time inferred as non-wear time contributed to the false positives (FP). The FPs, TPs, FNs, and TNs were calculated by looking at 10 s intervals of the acceleration data and comparing the inferred classification with the ground truth labels. From this we created a confusion matrix and derived overall accuracy as $\frac{TP + TN}{{TP + TN + FP + FN}}$, sensitivity as $\frac{TP}{{TP + FN}}$, precision as $\frac{TP}{{TP + FP}}$, and F1-score as $\frac{2TP}{{2TP + FP + FN}}$ to evaluate the classification performance of each non-wear detection technique. F1-score is the harmonic mean of precision and sensitivity with high F1-scores indicating good classification performance. Finally, we investigated the permutation predictor importance to understand the reason of better performances for each of the decision tree models.

For all analyses and model development, we used R version 4.1.2 (Bird Hippie) and RStudio version 2021.9.1.372 (Ghost Orchid) including the Tidymodels^24^ suit of packages for machine learning tools, and the package rpart^25^ as engine for the decision tree algorithm.

## Results

**Figure 3:**
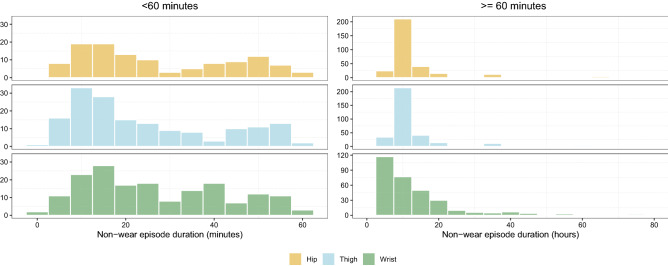
Distribution of the length of the non-wear episodes across hip, thigh, and wrist data. Distributions are shown for episodes shorter than 60 min and longer than 60 min.

In total across the three different wear locations, 1598 episodes of non-wear time were present in our gold standard datasets. The majority of these were non-wear episodes lasting ≥ 60 min, which accounted for 1148 (71.8%) episodes and had a mean duration of 794 min (SD = 1142), or approximately 13 h. Non-wear episodes lasting ≤ 60 min accounted for 450 (28.2%) of the episodes and had a mean duration of 26.4 min (SD = 16.4). Moreover, the shortest episodes (< 60 min) only constitute on average 1.3% of the total non-wear time across the three different wear-locations (see Table 2). Figure 3 shows the frequency distribution of episodes lasting < 60 min, and episodes lasting ≥ 60 min. The distribution of the short episodes of the PHASAR dataset was bimodal while the episodes longer than 60 min were most frequent around 10 h. The short episodes of the in-house dataset from wrist-worn devices were uniform whereas the long episodes were highly right skewed.

**Table 2:** Overview of non-wear episodes grouped in short and long non-wear episodes.

Wear location	Mean^1^	Cumulated^1^	Proportion^2^ (%)
< 60 min			
Hip	28	3202	1.13
Thigh	25	3975	1.40
Wrist	27	4691	1.32
≥ 60 min			
Hip	828	279,785	98.87
Thigh	776	280,294	98.60
Wrist	782	351,179	98.68

^1^Aggregated in minutes. ^2^Proportion of total non-wear time by wear location.

### Classification performance

**Figure 4:**
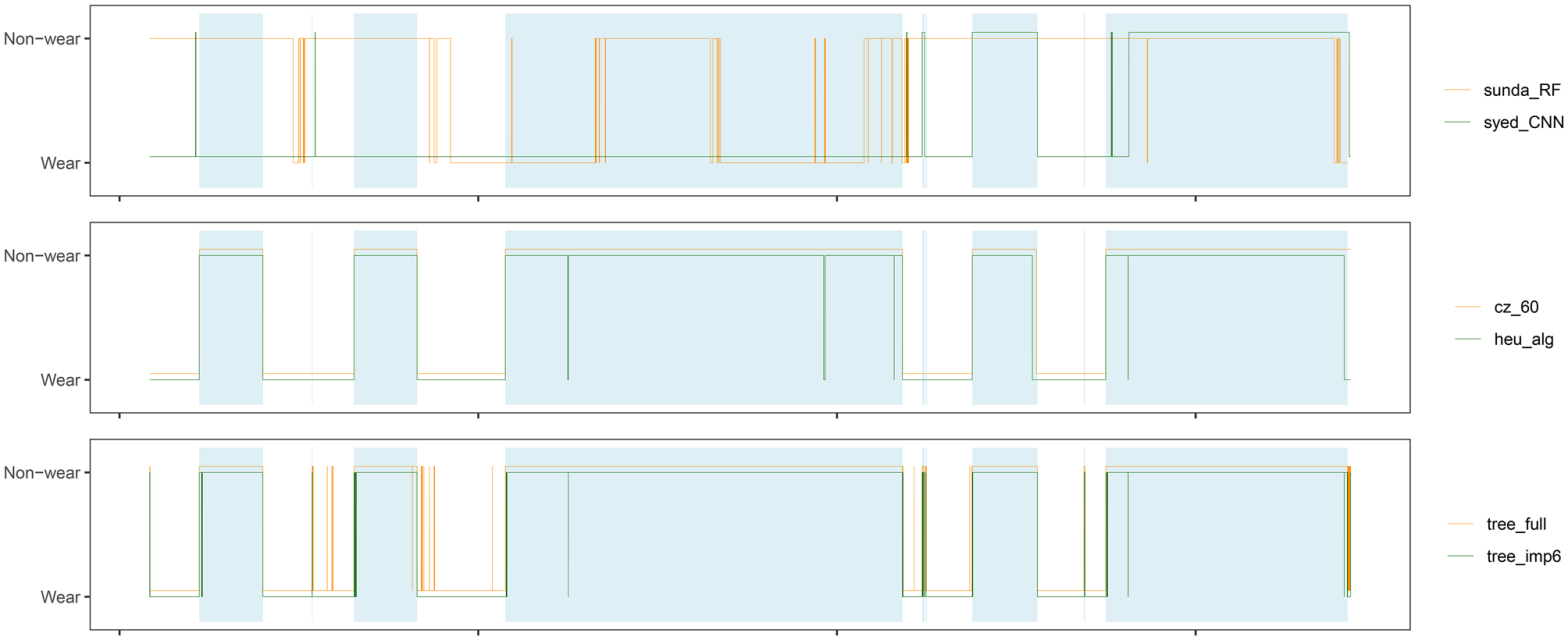
Visual example of the output of non-wear detection models and algorithms for a random person from the in-house wrist dataset (14 consecutive days). The light blue shade is ground-truth non-wear time. *Syed_CNN*, *cz_60*, and *tree_full* are vertically offset for easier interpretation.

Figure 4 shows an illustrative example of the outputs from the machine learned models and the rule-based algorithms shown comparatively with the ground truth non-wear time as shaded light blue background color. This particular case displays the general trend that the tree-based models are accurate but also volatile whereas the threshold-based methods (i.e., *Syed_CNN*, *heu_alg* and *cz_60*) are more stable. It is also evident in this recording, that the simple *cz_60* and *heu_alg* algorithms are not able to capture the short episodes.

**Figure 5:**
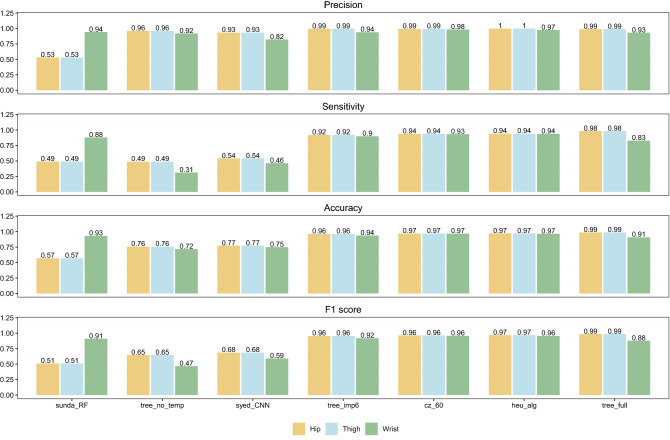
Classification performance metrics on all non-wear episodes for the seven included methods for classifying non-wear time. Metrics are shown for the three different ground-truth dataset including hip-worn, thigh-worn, and wrist-worn raw accelerometer data.

**Figure 6:**
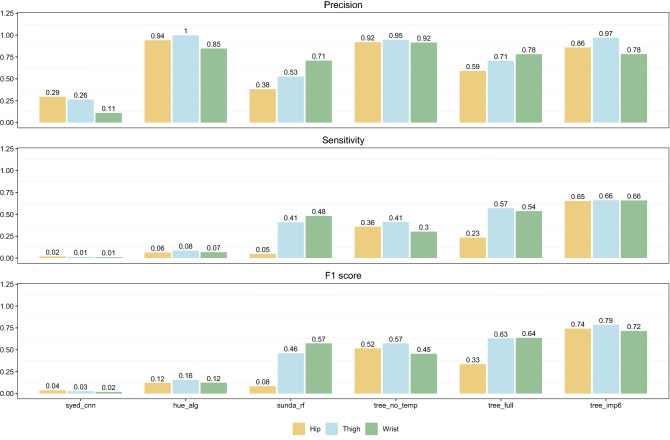
Classification performance for episodes no longer than 60 min in length. Metrics are shown for the three different gold-standard dataset including hip-worn, thigh-worn, and wrist-worn raw accelerometer data.

Figure 5 summarizes all classification performance metrics for all methods included in this study. The CNN model by Syed et al. performed similar across all three datasets with an overall accuracy ranging from 75% to 80%. The CNN provides the highest sensitivity score with 93% to 96%. Furthermore, F1 scores ranging from 82% to 84% were obtained by the CNN which was impaired by a mediocre precision score. The random forests by Sundararajan et al. performed the best on the wrist data having an F1 score of 94% and accuracy of 93% but otherwise performed poorly on the hip and thigh data in comparison to the other methods with overall accuracy scores 56% and precision scores of 59% indicating the presence of many false positives (the misclassification of true wear time as non-wear time). The decision tree model without the surface skin temperature as a predictor was the worst performing of the decision tree models on the wrist data having an overall accuracy score of 72%. Although the model obtained an excellent sensitivity score of 98%, the poor precision of the model resulted in a lower F1 score of 81% compared to both the CNN and the random forests. The remaining two decision tree models, including the six most important predictors and the full model, scored excellent across all performance metrics and across all three datasets. Finally, the *heu_alg* and the *cz_60* algorithms scored near perfect across all metrics and datasets. Performance metrics on episodes less than or equal to 60 min in length are shown in Fig. 6. As expected, the results show that the simple consecutive zeros algorithm detects no non-wear (not depicted in Fig. 6). The deep learning model by Syed et al. performed poorly across all metrics only detecting 1–2% of all non-wear time resulting in F1 scores below 5%. The *heu_alg* algorithm elicited high precision scores but combined with poor sensitivity score the resulting F1 scores ranged from 12% to 16% across the different wear locations. The random forest model performed mediocre on thigh and wrist data, and poor on the hip data with F1 scores of 46%, 57% and 8%, respectively. out of the three decision tree models, the model including the six most important predictors performed the best with decent F1 scores ranging from 72% to 79%. The decision tree model trained on all predictors (*tree_ful*l) performed poorly on the hip data due to a low sensitivity score of 23%. The decision tree trained on all predictors, but surface skin temperature showed excellent precision but because of low sensitivity, the F1 scores ranged from 45% to 57%.

## Discussion

Based on our results, the simplest methods (i.e., *cz_60* and *heu_alg*) for classifying non-wear episodes of lengths longer than 60 min performed excellent across all three wear locations closely followed by the decision tree models which included the surface skin temperature as a predictor. The random forest model also performed excellent on the wrist while the performance on the hip and thigh were mediocre. The deep learning and decision tree models without surface skin temperature as a predictor performed mediocre across all three sensor wear locations. As expected, when only examining the short non-wear episodes (< 60 min), the *cz_60* and the *heu_alg* algorithms were per default limited by their minimum episode duration of 60 and 20 min, respectively; thus, they performed poorly. The deep learning model performed poorly mainly due to a low sensitivity score resulting in many episodes falsely classified as non-wear. The random forest model performed poorly on the hip and mediocre on the thigh and wrist. We also observed mediocre performance for the decision tree model without temperature and the decision tree including all predictors. The best performing model on the short non-wear episodes was the decision tree trained on the six most important predictors. Lastly, inclusion of surface skin temperature to increase the predictive performance of non-wear time is supported by the results of this study.

The vast majority of the non-wear episodes in our ground truth datasets were longer than 60 min in length. In fact, the distribution of episode lengths revealed a peak around 10 h of duration which may be atypical compared to previous findings that show shorter episodes are the most numerous^10,22,26^. The characterization of the non-wear episodes within our data seems to favor the simple heuristic approaches for classifying non-wear time as the proportion of non-wear time lost to the limitations of employing a minimum window length are minuscule. Furthermore, the simple algorithms were able to obtain excellent precision scores indicating that no sedentary time or sleep was misclassified as being non-wear time which contrasts with several previous studies that have highlighted the difficulties performing this distinction^9,14,19,27–30^. Indeed, the present study included children and physically active adolescents which are known to spend less time sedentary and breaking up sedentary time more frequently^31,32^. This may accentuate the distinction between sedentary behavior and non-wear time suggesting that using a consecutive zeros algorithm may be considered best practice to capture non-wear episodes lasting longer than 60 min in children and adolescents across the wear locations included in this study, i.e., hip, thigh, and wrist. In addition, it is evident that the differences in physical activity behavior between children/adolescents and older adults will limit the ability to generalize models trained on specific age groups to other age groups. However, this may be less applicable regarding the standardized procedure of mounting and unmounting the accelerometer, as is the case with the *syed_CNN* model.

The task of creating a model to classify non-wear time can be considered simple. The underlying decision boundary is likely close to linear; and thus, the more complex models included in the current study may end up over-fitting to the random variation that are present only in the specific dataset used for training which deteriorates the generalizability to unseen data. Therefore, we speculate that an adequately optimized logistic regression model would likely perform on par with the included methodologies as the separating linear hyperplane would be able to differentiate between wear and non-wear time. Thus, utilizing highly non-linear models for the classification of non-wear time may be unnecessarily complex if the goal is to create a machine learning model that is to be employed across various populations and wear sites. Alternatively, it is essential to include different wear locations and a variety of physical activity profiles in the training data.

To the best of our knowledge, including the surface skin temperature for the classification of non-wear time has only scarcely been investigated using machine learning techniques with a single study showing that acceleration in combination with rate-of-change in surface skin temperature can result in a robust decision tree model for the detection of non-wear time^33^. Previous studies have incorporated temperature in heuristic non-wear algorithms showing that predictive performance is to be gained^14,16^ which is in accordance with our results that clearly demonstrate that including surface skin temperature improves the performance of the non-wear model. A critical aspect is the exact detection of the non-wear/wear onset and the slow temperature step response time of the sensor. Relying solely on temperature may introduce potential delays in the classification if the response time of the temperature sensor is slow whereas combining both temperature and acceleration data seems to be preferable^16^. We observed a 20 min step response of the Axivity temperature sensor, which might be explained by the way the devices casing has been designed. Additionally, the temperature is also dependent on the type of attachment method employed. When more material is positioned between the skin and the device, the longer the delay will be; thus, the machine learned models might ought to take type of sensor attachment into account. Moreover, device brands are likely to influence the temperature data collection as different temperature thresholds have been found to be optimal for distinct brands of devices, and as pointed out by Duncan et al. and Zhou et al. modifications were needed for the algorithms to work in different latitudes^14,16^.

Processing accelerometry data, it would be preferable to use the same model across various wear locations and populations, hence; to further qualify the robustness of the generalization performance of the included methods in the present study, we incorporated a dataset from wrist worn devices. By introducing a dataset from wrist-worn devices, we ensure the performance metrics of our developed decision tree models are not inflated due to overfitting because of lack of variance between training and testing data. This procedure is known as *external validation* and involves using independently derived datasets to validate the performance of a model trained on initial input data. Due to the test dataset coming from an independent source, any predictor set that may have been falsely selected due to characteristics of the input training data (e.g., technical or sampling bias) would likely fail. Hence, a positive performance in external validation is regarded as a proof of generalizability^34^. The logic behind external validation is sound: data taken from separate sources have less in common, but nonetheless may capture useful domain-relevant aspects. A well-trained model that captures informative predictors is robust and will continue to exhibit good results even when repeatedly challenged with new data. The external validation in the current study; thus, provides an assurance that our developed decision tree models passing this step are more likely domain interpretable^35^.

Although the methodology by Syed et al. is innovative and follows a clear behavioral logic, we speculate that the signal shape of the raw acceleration right before and right after a candidate non-wear episode may be dependent on population age. Methodologies designed to identify non-wear time by the absence of acceleration are by definition independent of population characteristics as the collected data during non-wear is zero movement. The method proposed by Syed et al. characterizes non-wear by identifying the acceleration signal shape that marks the beginning and end of a non-wear episode. Thus, analyzing the physical activity behavior of the population and not the absence of acceleration, which might be more dependent on population characteristics. Thus, the poor performance seen in the present study by the CNN model by Syed et al. may be due to variations in the dataset populations. The CNN model was trained on data from an older population aged 40–84 years (mean = 62.74; SD = 10.25) compared to the datasets employed in the present study, aged 8.1–17.9 years (mean = 12.14, SD = 2.40) from the hip and thigh data, and aged 14.5–16.4 years old (mean = 15.4, SD = 0.37 years) from the wrist data. This is supported by our results showing that the *sunda_RF* model performs acceptable when identifying non-wear episodes shorter than 60 min on thigh and wrist data whereas *syed_CNN* performs poorly across all wear locations. This indicates that the model by Sundararajan et al. seem to be population characteristic agnostic as expected in contrast to the *syed_CNN* model. Finally, it is important to note that the *syed_CNN* model was originally trained on data with a frequency of 100 Hz. In our current study, we have used the model on data with frequencies of 50 Hz and 25 Hz. It is not clear if this difference in data frequency has had any effect on the model's performance. However, as movement frequencies is generally below 5 Hz we are confident that the 25 Hz data is sufficient to capture the true movement behavior for the subject mounting or unmounting the device.

It is customary practice to report performance of a model on a test split from the data that was used to train the model. This is called *internal validation* and is a sensible approach, but this also entails a minimum of variation between the train and test data. While the reported metrics, such as sensitivity, specificity and/or accuracy for the classification of non-wear time, are reported to be remarkably high by Syed et al.^18^ and Sundararajan et al.^17^ these results are obtained via cross-validation without an external validation dataset, which weakens the confidence of the generalizability of these models. Highly flexible models potentially overfit the training data distribution when independent test sets are not used or are prone to learn dataset-specific artifacts rather than more generalizable behavioral characteristics. As a rule of thumb, every machine learning and deep learning approach would benefit from larger training datasets, although we acknowledge that this is rarely practically feasible. However, this may be particularly true for the model developed by Syed et al. Thus, to make their methodology more robust, the training data employed would benefit from a more diverse population and a higher number of participants as the varying signal shapes related to the mounting and un-mounting of the device may be dependent across age groups and other population characteristics. Therefore, for future developments within this area, we encourage the practice of validating performance on independent external datasets prior to publishing the model.

The main strength of the study is the use of external validation, as this is considered good evidence of method generalizability. One limitation is the construction of our ground truth datasets. As no accepted gold-standard exists within this field of research, ground truth estimates of non-wear vary across studies, thus hindering our ability to compare performance metrics. However, by utilizing raw accelerometer data, our methods are transparent, and no intermediate steps of the data collection and analysis are proprietary. Moreover, the results are based on a population of children and adolescents, and as such, cannot be generalized to older populations. Finally, other machine learning algorithms may be preferable, however; we chose to build a decision tree model as the complexity of this models still makes it possible to make meaningful interpretations. More research is needed to determine the effectiveness of other methods, i.e., logistic regression, gradient boosting, support vector machines and others.

## Conclusions

In this study we present results on the performance and generalizability of existing methods and newly developed decision tree models for the classification of non-wear in free-living accelerometer recordings. Although the current available heuristic methods have shown promising results, they are subject to obvious limitation whereas recent complex machine learning methods may be prone to over-fitting as our results indicate. Furthermore, the quantity and quality of data are essential when training a machine learning model for a simple binary classification problem when researchers want to be able to generalize to other types of data. For this, we encourage the use of external validation to temper overoptimistic expectations of model performance in unseen data. Furthermore, for the crucial first step when analyzing accelerometer data (i.e., detecting non-wear time), we advise researchers to carefully select a proper method for this task.

## Data Availability

The classification models developed in this paper are available on request to the corresponding author as well as the raw data from the PHASAR study and the raw data from the in-house validation study.
